# Therapeutic potential of crude protein extracts from two Egyptian freshwater snails *Lanistes carinatus* and *Bellamya unicolor*

**DOI:** 10.1038/s41598-026-60044-5

**Published:** 2026-07-04

**Authors:** Mohamed R. Habib, Azza H. Mohamed, AbdElhafez R. AbdElhafez, Mohamed S. Helmy, Ahmed A. Hamed, Aya I. F. Akila, Hesham R. El-Seedi, Shaden A. M. Khalifa, Hassan M. Masoud

**Affiliations:** 1 Medical Malacology Department, Theodor Bilharz Research Institute, 1 Corniche El Nile St., Warrak El-Haddar, Imbaba, Giza, 12411 Egypt; 2 Zoology Department, Faculty of Science, Menoufia University, Shebin El-Kom, Egypt; 3 Molecular Biology Department, National Research Centre, 33 El-Buhouth St., Dokki, Giza, 12622 Egypt; 4 Proteome Research Lab, Central Laboratories Network and Centers of Excellence, National Research Center, 33 El-Buhouth St., Dokki, Giza, 12622 Egypt; 5 Microbial Chemistry Department, National Research Center, 33 El-Buhouth St., Dokki, Giza, 12622 Egypt; 6 Department of Chemistry, Faculty of Science, Menoufia University, Shebin El-Kom, 31100107 Egypt; 7 Neurology and Psychiatry Department, Capio Saint Göran’s Hospital, Sankt Göransplan 1, 112 19 Stockholm, Sweden

**Keywords:** Freshwater snails, *Lanistes carinatus*, *Bellamya unicolor*, Antioxidant enzymes, Antimicrobial activity, Antibiofilm activity, Cytotoxicity, Bioactive peptides, Biochemistry, Biotechnology, Drug discovery, Microbiology

## Abstract

Freshwater snails are emerging sources of bioactive molecules with potential biomedical and therapeutic relevance. This study evaluated crude protein extracts from two Egyptian freshwater snails, *Lanistes carinatus* and *Bellamya unicolor*, for antioxidant enzymes, antimicrobial and antibiofilm activities, cytotoxicity against human cancer cell lines, and peptide composition. Both species exhibited low activities of superoxide dismutase (SOD), catalase (CAT), and glutathione S-transferase (GST), with slightly higher levels in *L. carinatus*. Despite this, the crude protein extracts inhibited the growth of *Escherichia coli* and *Staphylococcus aureus*; *L. carinatus* showed stronger antifungal activity against *Candida albicans*, while neither extract affected *Aspergillus niger*. Selective cytotoxicity was observed*: L. carinatus* was most active against MCF-7 and HeLa cells, whereas *B. unicolor* was highly potent against HCT-116 and moderately active against PC3 cells, with minimal effects on normal WI-38 cells. LC–MS/MS identified 26 short peptides, likely contributing to the antimicrobial and anticancer activities.These findings provide preliminary evidence of the therapeutic potential of *L. carinatus* and *B. unicolor* crude protein extracts, highlighting freshwater snails as candidate sources of bioactive molecules that merit further investigation.

**Supplementary Information:**

The online version contains supplementary material available at 10.1038/s41598-026-60044-5.

## Introduction

Mollusks represent one of the most diverse animal phyla and have attracted growing interest as sources of bioactive compounds. Gastropod snails, in particular, produce a wide variety of proteins, peptides and secondary metabolites with antioxidant, antimicrobial and anticancer properties^1,2^. Antioxidant enzymes such as superoxide dismutase (SOD) and glutathione S-transferase (GST) are central to the detoxification of reactive oxygen species and help protect cellular macromolecules from oxidative damage, thereby contributing to immune defense and cancer prevention^3,4^.

Beyond antioxidant enzymes, snails synthesize bioactive molecules that include antimicrobial peptides, lectins and cytotoxic proteins. These molecules can inhibit the growth of bacteria and fungi, modulate immune responses and interfere with the survival of tumor cells^5–7^. Terrestrial snails such as *Cornu aspersum* and *Eremina desertorum* secrete mucus rich in peptides and glycoproteins with notable antibacterial, antifungal and anticancer actions^8,9^. Marine mollusks are also recognized for potent bioactive products, including protein hydrolysates with immunostimulatory and antitumor effects^5^ and lectins from sea-hare eggs with antiproliferative activity^6^.

Freshwater snails represent another important but comparatively less explored group. In Egypt, *Biomphalaria alexandrina*, *Bulinus truncatus*, *Helisoma duryi*, *Lanistes carinatus* and *Bellamya unicolor* are widely distributed in the Nile Delta and associated waterways^10^. Some of these freshwater snails act as intermediate hosts of schistosomes and thus have been extensively studied from a parasitological perspective^11^. At the same time, several studies have revealed that freshwater snails harbor antioxidant and antimicrobial constituents with potential therapeutic value. For example, tissues of the freshwater snail *Brotia costula* showed moderate antibacterial and antifungal activity and contained nutritionally valuable proteins and lipids, while crude protein extract of *Lanistes carinatus* enhanced the immune response of *B. alexandrina* and reduced susceptibility to *Schistosoma mansoni* infection^11,12^.

Increasing attention has also been paid to snail-derived peptides. Peptidomic analysis of mucus from the terrestrial snail *Achatina fulica* predicted several peptide sequences with potential anticancer activity against breast cancer cells^7,14^. Comparative proteomic and peptidomic studies of snail mucus revealed that each species possesses a characteristic set of small peptides, some of which may have antimicrobial or signaling functions^15^. Freshwater snails, however, remain less characterized in terms of their peptide repertoires and the relationship between these peptides and biological activities.

*Lanistes carinatus* (family Ampullariidae) and *Bellamya unicolor* (family Viviparidae) are two freshwater snails common in Egyptian aquatic ecosystems. They share habitats with medically important schistosome-transmitting snails but are not themselves known to act as intermediate hosts^10^. These non-schistosome snails provide a useful system in which to explore bioactive compounds without confounding effects of parasite–host interactions. Previous toxicological work has shown that *L. carinatus* responds to pollutants such as chlorpyrifos through antioxidant and stress-related pathways^16^, suggesting that its tissues may contain defensive molecules of potential biomedical relevance.

The present study aimed to evaluate crude protein extracts from *L. carinatus* and *B. unicolor* as sources of natural antioxidant enzymes, antimicrobial agents and cytotoxic molecules. Specifically, we (i) quantified SOD, catalase (CAT) and GST activities in the crude protein extracts; (ii) compared overall protein profiles using sodium dodecyl sulfate–polyacrylamide gel electrophoresis (SDS–PAGE); (iii) assessed antibacterial, antifungal and antibiofilm activities against selected bacterial and fungal pathogens; (iv) evaluated cytotoxicity against a panel of human cancer cell lines and normal fibroblasts, comparing the results with doxorubicin; and (v) characterized low-molecular-weight peptide constituents using liquid chromatography–tandem mass spectrometry (LC–MS/MS). By integrating biochemical, microbiological, cytotoxic and peptidomic data, this work investigates the bioactive potential of two common Egyptian freshwater snails and highlights their possible relevance as candidate sources for future nutraceutical and pharmaceutical development.

## Materials and methods

### Snail collection and protein extraction

The freshwater snails *L. carinatus* and *B. unicolor* investigated here are non-protected, lower invertebrate species and were collected from a freshwater locality at Lake Manzalah, Dakahlia Governorate, Egypt (31° 11′ N, 32° 02′ E). This study was conducted at the Medical Malacology Department, Theodor Bilharz Research Institute, as part of a field-based survey on species identification, disease transmission potential, and bioactive molecules. Under prevailing regional guidelines and institutional policies, the collection and use of non-endangered freshwater invertebrates for survey-based or exploratory research do not require formal approval from an animal ethics committee or specific permits. All sampling was performed following standard field practices, ensuring minimal environmental disturbance and responsible specimen handling. Ten adult snails (Shell-size ranges were 20–30 mm for *L. carinatus*, and 18–25 mm for *B. unicolor*) were used from each species. Snails were maintained alive in well-aerated tap water at approximately 20 °C and fed fresh lettuce until use. Soft tissues were dissected after shell removal, rinsed in cold saline to remove debris and stored at − 20 °C until extraction.

Crude protein extracts were prepared with slight modifications of standard protocols. Frozen whole soft tissues (2–3 g wet tissue from each species-specific pooled sample) were thawed on ice and homogenized (1:3 w/v) in 20 mM potassium phosphate buffer (pH 7.0). Homogenates were centrifuged at 10,000 × g for 30 min at 4 °C to remove insoluble material^2^. The supernatant was frozen and subsequently freeze-dried to obtain a dry protein powder. The lyophilized material was then reconstituted in an appropriate volume of 20 mM potassium phosphate buffer (pH 7.0) prior to further analyses. Excess salt was removed by overnight dialysis against the same buffer. The desalted crude protein extract was stored at 4 °C and its protein concentration determined using the Bradford assay with bovine serum albumin as a standard^17^.

### Antioxidant enzyme assays

Activities of catalase, superoxide dismutase and glutathione S-transferase were measured spectrophotometrically. CAT activity was determined using a reaction mixture (3.0 mL) contained 0.05 M potassium phosphate buffer (pH 7.0) and 0.02 M hydrogen peroxide (H_2_O_2_). The reaction was initiated by addition of enzyme solution and the decrease in absorbance at 240 nm due to H_2_O_2_ decomposition was recorded for 1 min. One unit of CAT activity was defined as the amount of enzyme required to decompose 1 µmol of H_2_O_2_ (using an extinction coefficient of 43.6 M^−1^cm^−1^) per minute at 25 °C^18^.

SOD activity was measured in a 1.0 mL reaction mixture contained 0.05 M potassium phosphate buffer (pH 7.8), 0.01 mM cytochrome c, 0.1 mM EDTA and 0.05 mM sodium xanthine. The reaction was started by adding xanthine oxidase (21 mU), and the reduction of cytochrome c was monitored at 550 nm. One unit of SOD activity was defined as the amount of enzyme causing 50% inhibition of cytochrome c reduction^19^.

GST activity was assayed in a 1.0 mL mixture contained 0.1 M potassium phosphate buffer (pH 6.5), 1 mM reduced glutathione (GSH), 1 mM 1-chloro-2,4-dinitrobenzene (CDNB) dissolved in ethanol (final ethanol < 4%) and the enzyme solution. The formation of the GSH–CDNB conjugate was followed as an increase in absorbance at 340 nm at 37 °C for 3 min, using an extinction coefficient of 9.6 mM^−1^cm^−1^. One unit of GST activity was defined as the amount of enzyme catalyzing the conjugation of 1 µmol of CDNB per minute^20^. All measurements were carried out in triplicate and expressed as mean ± standard deviation (SD).

### SDS–PAGE protein profiling

Protein profiles of *L. carinatus* and *B. unicolor* crude protein extracts were examined by SDS–PAGE. Samples were mixed with Laemmli sample buffer containing SDS and β-mercaptoethanol, heated at 95 °C for 5 min and loaded onto 5% stacking / 12% resolving polyacrylamide gels. Electrophoresis was performed at constant voltage following Laemmli (1970), using pre-stained molecular mass markers for calibration^21,22^. Gels were stained with Coomassie Brilliant Blue R-250 and destained until discrete bands were visible. Apparent molecular masses of major bands were estimated by comparison with the markers.

### Antimicrobial activity

The antimicrobial activity of the crude protein extracts was evaluated at 500 µg/mL. Aliquots of 50 µL were loaded onto 5 mm sterile filter-paper discs (Whatman No. 1), dried and placed on agar plates seeded with the test microorganisms. Bacterial and yeast cultures were prepared on nutrient agar, whereas fungal cultures were grown on potato dextrose agar (PDA; DSMZ 130). Microbial suspensions were adjusted to 10^7^–10^8^ CFU/mL. Plates were incubated at 37 °C for 24 h for bacteria and yeast and at 30 °C for 48 h for fungi. Inhibition zones were measured in millimetres. The test organisms were *Escherichia coli*, *Staphylococcus aureus*, *Candida albicans* and *Aspergillus niger*^23^. Ciprofloxacin and nystatin served as the antibacterial and antifungal positive controls, respectively, while the protein buffer solution served as the negative control. All strains were obtained from the culture collection of the Microbial Chemistry Department, National Research Centre, Egypt.

The minimum inhibitory concentration (MIC) of the crude protein extracts was determined in 96-well flat-bottom polystyrene microplates. Serial two-fold dilutions of the extracts were prepared in lysogeny broth (LB) at final concentrations of 500, 250, 125, 62.5, 31.25, 15.62, 7.81, 3.90 and 1.95 µg/mL in a total volume of 150 µL per well. Each well was inoculated with 10 µL of a logarithmic-phase bacterial culture and incubated overnight at 37 °C. Bacterial growth inhibition was assessed visually (clear wells indicating inhibition and turbid wells indicating growth) and quantitatively by measuring the absorbance at 600 nm (OD_600_) after approximately 20 h using a SpectroStar Nano microplate reader (BMG LABTECH GmbH, Allmendgrün, Germany). Untreated bacterial cultures served as controls, and the MIC was defined as the lowest crude protein extract concentration that produced no visible growth.

### Biofilm inhibitory activity

The biofilm inhibitory activity of the crude protein extracts was evaluated using the microtiter-plate (MTP) assay against clinical strains of *Pseudomonas aeruginosa*, *Staphylococcus aureus*, *Escherichia coli* and *Bacillus subtilis*^24^. Biofilm formation was assessed in 96-well flat-bottom polystyrene plates by adding 180 µL of LB broth (10 g tryptone, 5 g yeast extract and 10 g NaCl per litre) and 10 µL of an overnight bacterial culture per well, followed by 10 µL of crude protein extract. Plates were incubated at 37 °C for 24 h, washed with phosphate-buffered saline (PBS, pH 7.2) to remove non-adherent cells, and the remaining sessile bacteria were fixed with 2% sodium acetate and stained with 0.1% crystal violet. After washing and air-drying, the bound dye was solubilised with 95% ethanol and the absorbance was measured at 595 nm using a microplate reader (BMG LABTECH GmbH, Allmendgrün, Germany). A blank control (growth medium without extract) served as the baseline for calculating the percentage of biofilm inhibition. The assay was performed in triplicate and the results are expressed as mean ± SD.

### Cytotoxicity assay

Cytotoxicity of the crude protein extracts was assessed with the MTT assay against HepG2 hepatocellular carcinoma, MCF-7 breast adenocarcinoma, HCT-116 colorectal carcinoma, PC3 prostate carcinoma, HeLa cervical carcinoma and WI-38 normal human lung fibroblasts. Cell lines were obtained from ATCC via VACSERA (Cairo, Egypt). Doxorubicin was used as a standard chemotherapeutic control.

Cells were cultured in RPMI-1640 medium supplemented with 10% fetal bovine serum, 100 U/mL penicillin and 100 µg/mL streptomycin at 37 °C in a humidified 5% CO_2_ atmosphere. Cells were seeded in 96-well plates at 1 × 10^4^ cells per well and allowed to adhere for 48 h, then treated with serial concentrations of crude protein extracts or doxorubicin for 24 h. After treatment, 20 µL of MTT solution (5 mg/mL) were added to each well and incubated for 4 h. Medium was removed and 100 µL of dimethyl sulfoxide were added to dissolve formazan crystals. Absorbance was recorded at 570 nm, and cell viability was calculated as (A₅₇₀ treated/A₅₇₀ control) × 100. IC_50_ values were obtained from dose–response curves^25,26^.

### LC–MS/MS peptide analysis

Low-molecular-weight fractions of *L. carinatus* and *B. unicolor* crude protein extracts were analyzed by LC–MS/MS^27,28^. Samples were injected onto a narrow-bore C18 column coupled to an electrospray ion trap mass spectrometer operated over an m/z range of 200–5000. Mobile phases consisted of 0.1% formic acid in water (A) and 0.1% formic acid in acetonitrile (B). A gradient from 5 to 95% B was run over 40 min at 300 µL/min, followed by re-equilibration to 5% B.

Raw data were converted to mzXML format using MSConvert (ProteoWizard), and peaks were processed with MZmine. Peptides were identified by matching precursor masses and MS/MS fragmentation patterns to those previously reported for snail mucus peptides^15^. Retention times, sequences and fragment ions are summarised in Tables 4 and 5.

### Statistical analysis

All assays were performed as three independent biological replicates, each carried out in technical triplicate. For the disc-diffusion antimicrobial screening, the inhibition zones were recorded by direct physical measurement of the zone diameters; because this method yields discrete visual measurements rather than continuous quantitative readings, the number of replicates (*n* = 3) is reported rather than a standard deviation. The antibiofilm and MTT cytotoxicity assays were performed in triplicate, and their quantitative results are expressed as mean ± standard deviation (SD). IC_50_ values were determined from the dose–response curves by nonlinear regression analysis, and selectivity indices (SI) were calculated as the ratio of the IC_50_ for normal WI-38 fibroblasts to the IC_50_ for each cancer cell line. Data from enzyme activity tests as well as MTP and MTT assays were analyzed using GraphPad Prism software (version 8.1.1; GraphPad Software, San Diego, CA, USA). Differences among groups, including comparisons between the snail crude protein extracts and doxorubicin, were evaluated by one-way analysis of variance (ANOVA) followed by Tukey’s multiple-comparison test, with *p* < 0.05 considered statistically significant.

## Results

### Antioxidant enzyme activities

Activities of SOD, CAT and GST in crude protein extracts from *L. carinatus* and *B. unicolor* are presented in Table 1. For all three enzymes, *L. carinatus* showed slightly higher activities than *B. unicolor*. SOD activity reached 12.2 ± 1.8 U/mg protein in *L. carinatus* and 9.5 ± 2.2 U/mg protein in *B. unicolor*. Catalase activities were 1.5 ± 0.42 and 1.1 ± 0.36 U/mg protein, respectively. GST activity was low in both species but marginally higher in *L. carinatus* (0.18 ± 0.039 U/mg protein) than in *B. unicolor* (0.17 ± 0.043 U/mg protein). These data indicate that both freshwater snails possess modest enzymatic antioxidant capacity, with *L. carinatus* having a slightly stronger profile.

**Table 1:** Activities of superoxide dismutase (SOD), catalase (CAT) and glutathione S-transferase (GST) in crude protein extracts from *L. carinatus* and *B. unicolor.*

Enzyme	*L. carinatus* (U/mg protein)	*B. unicolor* (U/mg protein)
Superoxide dismutase (SOD)	12.2 ± 1.8	9.5 ± 2.2
Catalase (CAT)	1.5 ± 0.42	1.1 ± 0.36
Glutathione S-transferase (GST)	0.18 ± 0.039	0.17 ± 0.043

Activities were expressed as specific activities (U mg^−1^ protein) and presented as mean of triplicates ± standard deviation (SD).

### SDS–PAGE protein profiles

**Fig. 1:**
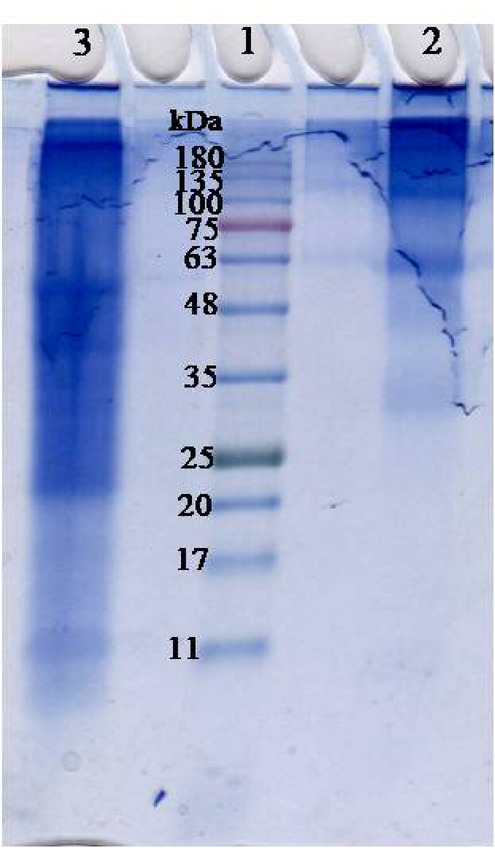
SDS–PAGE profiles of crude protein extracts from the freshwater snails *Lanistes carinatus* and *Bellamya unicolor*: Molecular weight markers (kDa) in lane 1, protein bands of *L. carinatus* in lane 2 and protein bands of *B. unicolor* in lane 3.

SDS–PAGE revealed species-specific protein banding patterns (Fig. 1). The *L. carinatus* crude protein extract showed several dominant bands between approximately 25 and 70 kDa, whereas the *B. unicolor* crude protein extract displayed one prominent band around 50 kDa and additional lower-molecular-weight bands below 20 kDa. These patterns suggest that each freshwater snail species has a characteristic set of abundant proteins, which may include structural proteins, enzymes and other bioactive components.

### Antimicrobial and antibiofilm activities

**Fig. 2:**
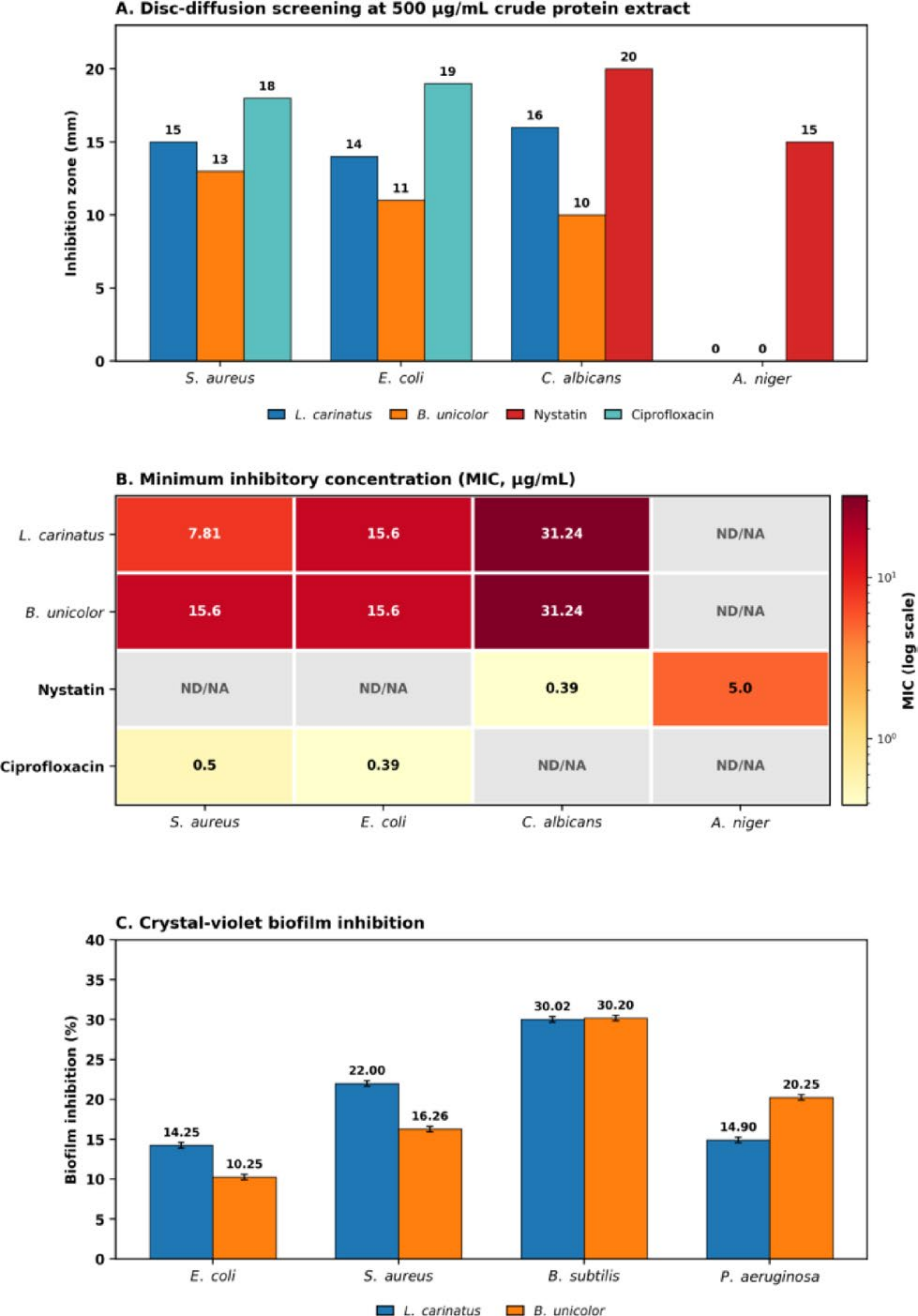
Antimicrobial activity of crude protein extracts from *L. carinatus* and *B. unicolor*. (**A**) Disc-diffusion inhibition zones (mm) at 500 µg/mL crude protein extract against *Staphylococcus aureus*, *Escherichia coli*, *Candida albicans* and *Aspergillus niger* for snail extracts and positive controls (ciprofloxacin, nystatin). (**B**) Minimum inhibitory concentrations (MICs, µg/mL) of the crude protein extracts against the same pathogens, with ciprofloxacin and nystatin included as reference antibiotics; ND/NA denotes not determined/not applicable. (**C**) Crystal-violet biofilm inhibition (%) produced by the crude protein extracts against the tested bacterial strains. Assays were performed in triplicate; values represent the percentage reduction in biofilm biomass relative to untreated controls.

Both snail crude protein extracts exhibited antibacterial and antifungal activities, although their potency differed among target organisms. In disc-diffusion assays, *L. carinatus* produced inhibition zones of about 14 mm against *E. coli* and 15 mm against *S. aureus*, while *B. unicolor* produced zones of approximately 11 mm and 13 mm, respectively (Fig. 2A). These zones were smaller than those produced by ciprofloxacin (18–19 mm) but clearly demonstrated moderate antibacterial activity.

For antifungal activity, *L. carinatus* crude protein extract showed a pronounced effect against *C. albicans* with an inhibition zone of about 16 mm, compared with ~ 10 mm for *B. unicolor*. Neither crude protein extract inhibited growth of *A. niger*, whereas nystatin produced zones of ~ 20 mm against *C. albicans* and ~ 15 mm against *A. niger* (Fig. 2A).

Minimum inhibitory concentration measurements supported these findings (Fig. 2B). The MIC of *L. carinatus* crude protein extract was 7.81 µg/mL against *S. aureus* and 15.6 µg/mL against *E. coli*, while *B. unicolor* showed an MIC of 15.6 µg/mL against both bacteria. Against *C. albicans*, both extracts had an MIC of 31.24 µg/mL, whereas neither extract inhibited *A. niger* (no MIC determined). As expected, the reference drugs were far more potent: ciprofloxacin gave MICs of 0.5 and 0.39 µg/mL against *S. aureus* and *E. coli*, and nystatin gave MICs of 0.39 and 5.0 µg/mL against *C. albicans* and *A. niger*, respectively.

In the antibiofilm assay, both crude protein extracts reduced biofilm formation by the tested bacteria (Table 2); all values are expressed as the mean ± SD of three independent replicates. At 100 µg/mL, *L. carinatus* crude protein extract decreased biofilm biomass by 30.02% for *B. subtilis* and 14.90% for *P. aeruginosa*, with smaller reductions for *E. coli* (14.25%) and *S. aureus* (22.00%). *B. unicolor* crude protein extract showed a similar pattern, with 30.20% inhibition for *B. subtilis*, 20.25% for *P. aeruginosa*, and lower inhibition for *E. coli* (10.25%) and *S. aureus* (16.26%). Although these percentages indicate moderate antibiofilm activity, they confirm that both freshwater snail crude protein extracts can interfere with surface-associated bacterial communities.

**Table 2:** Antibiofilm activity of protein extracts from *L. carinatus* and *B. unicolor* against selected bacteria (100 µg/mL).

Snail species	*E. coli* (%)	*S. aureus* (%)	*B. subtilis* (%)	*P. aeruginosa* (%)
*L. carinatus*	14.25 ± 0.35	22.00 ± 0.35	30.02 ± 0.35	14.90 ± 0.35
*B. unicolor*	10.25 ± 0.35	16.26 ± 0.35	30.20 ± 0.35	20.25 ± 0.35

Values represent percentage reduction in biofilm biomass relative to untreated controls.

### Cytotoxicity against cancer and normal cell lines

**Fig. 3:**
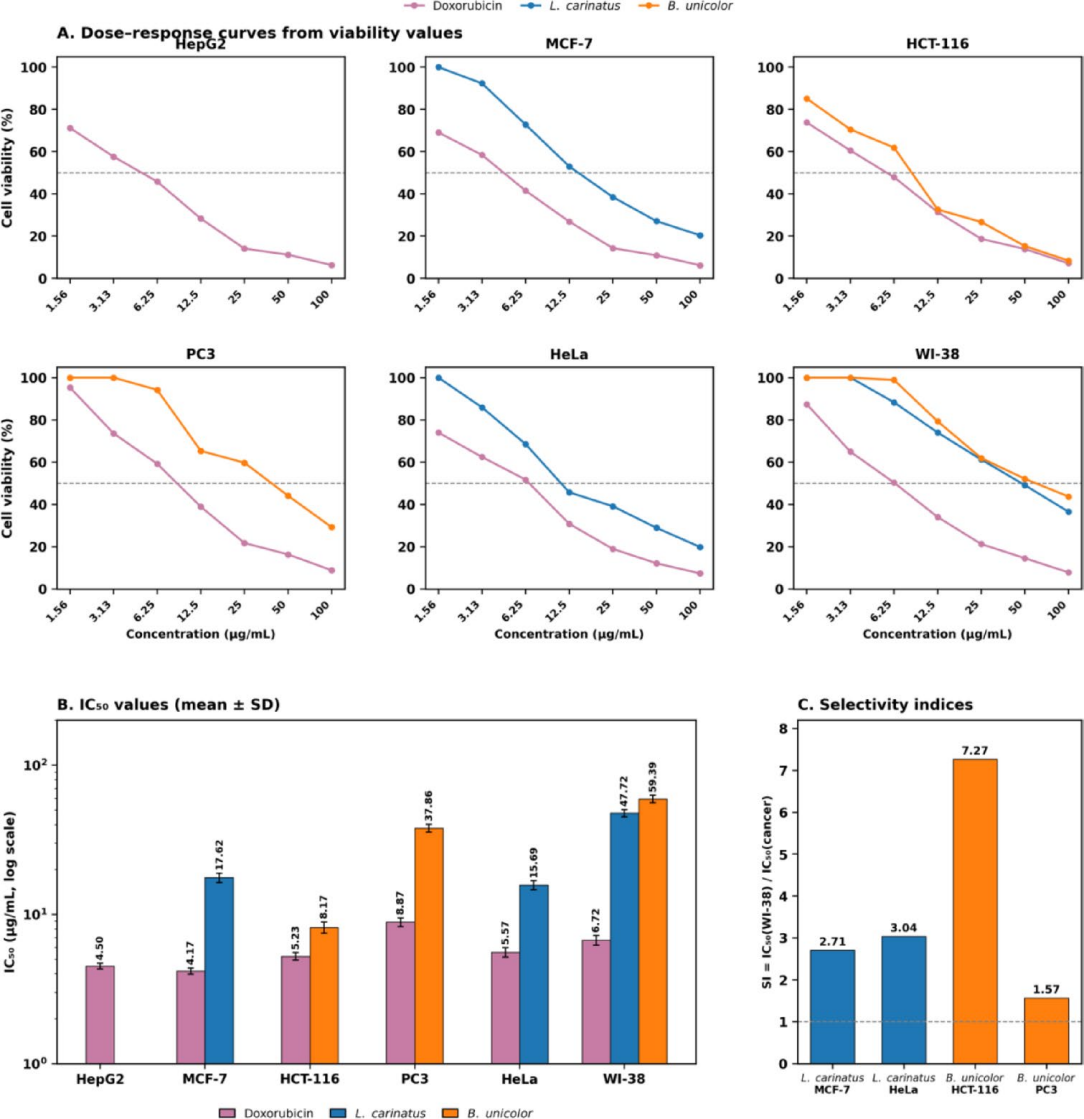
Cytotoxicity of crude protein extracts from *L. carinatus* and *B. unicolor* against human cancer and normal cell lines, with doxorubicin as a positive control. (**A**) Dose–response curves showing the relative viability of HepG2, MCF-7, HCT-116, PC3, HeLa and WI-38 cells treated with each agent at different concentrations; the dashed line indicates 50% viability. (**B**) Half-maximal inhibitory concentration (IC_50_) values (mean ± SD, µg/mL; log scale) for each agent across the six cell lines. (**C**) Selectivity indices (SI = IC_50_ of normal WI-38 fibroblasts ÷ IC_50_ of each cancer cell line); the dashed line marks SI = 1, above which an extract is more selective for cancer cells than for normal cells.

The snail crude protein extracts displayed distinct cytotoxic profiles across the tested cell lines. Overall IC_50_ values are summarized in Table 3 and illustrated in Fig. 3. *L. carinatus* showed strong cytotoxicity towards MCF-7 breast cancer cells (IC_50_ = 17.62 ± 1.3 µg/mL) and HeLa cervical cancer cells (IC_50_ = 15.69 ± 1.1 µg/mL), but did not reach 50% inhibition for HCT-116, PC3 or HepG2 within the tested concentration range (≤ 100 µg/mL). In contrast, *B. unicolor* crude protein extract was very potent against HCT-116 colorectal cancer cells (IC_50_ = 8.17 ± 0.7 µg/mL) and showed moderate activity against PC3 prostate cancer cells (IC_50_ = 37.86 ± 2.3 µg/mL), while its effects on MCF-7, HeLa and HepG2 were weak.

**Table 3:** IC_50_ values (µg/mL) of doxorubicin and snail protein extract against human cancer and normal cell lines. Doxorubicin was used as a positive control.

Compound	HepG2	MCF-7	HCT-116	PC3	HeLa	WI-38
Doxorubicin	4.50 ± 0.2	4.17 ± 0.2	5.23 ± 0.3	8.87 ± 0.6	5.57 ± 0.4	6.72 ± 0.5
*L. carinatus* extract	–	17.62 ± 1.3	–	–	15.69 ± 1.1	47.72 ± 2.6
*B. unicolor* extract	–	–	8.17 ± 0.7	37.86 ± 2.3	–	59.39 ± 3.3

IC_50_ values represent the concentrations required to inhibit 50% of cell viability and are expressed as mean ± SD, as determined from dose–response curves. “–” indicates IC_50_ > 100 µg/mL under the tested conditions.

Importantly, both crude protein extracts were much less toxic to normal WI-38 fibroblasts. IC_50_ values were 47.72 ± 2.6 µg/mL for *L. carinatus* and 59.39 ± 3.3 µg/mL for *B. unicolor*, placing them in the weak cytotoxicity range and indicating a favorable selectivity index compared with doxorubicin. Doxorubicin exhibited IC_50_ values of 4–9 µg/mL across all cancer cell lines and 6.72 ± 0.5 µg/mL for WI-38, reflecting its well-known lack of selectivity (Table 3, Fig. 3).

### Peptide identification by LC–MS/MS

**Fig. 4:**
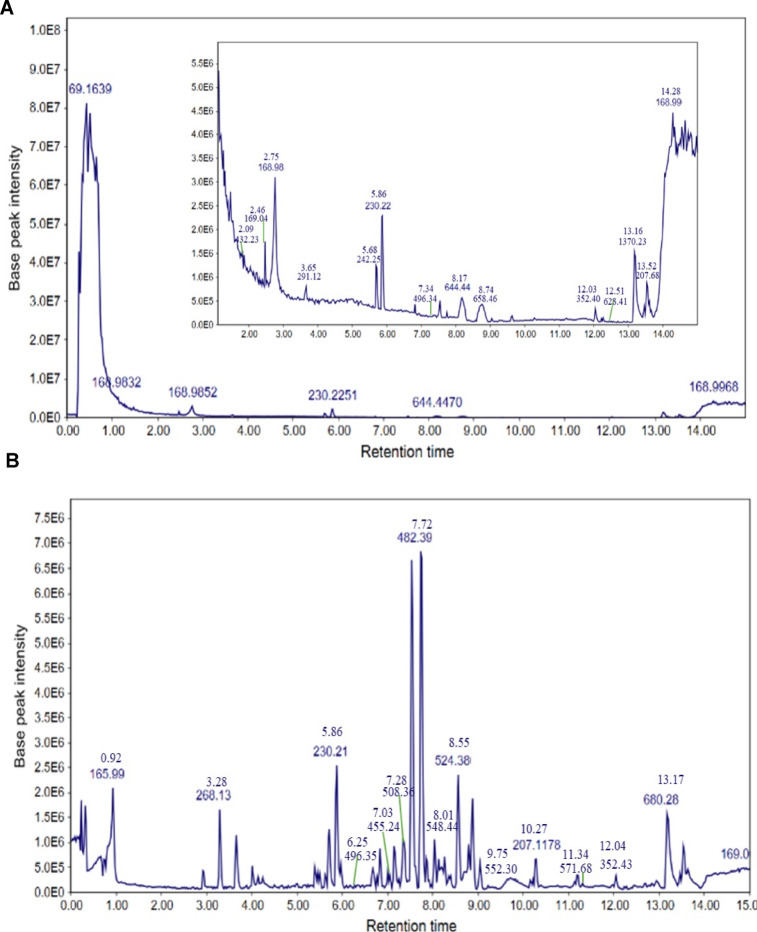
LC–MS/MS base-peak chromatograms of crude protein extracts from *L. carinatus* (**A**) and *B. unicolor* (**B**), showing major peptide peaks and corresponding m/z values.

LC–MS/MS analysis of low-molecular-weight fractions revealed multiple small peptides in both snail crude protein extracts. The base-peak chromatograms displayed several early-eluting peaks consistent with polar peptides (Fig. 4). In *L. carinatus*, most peptide peaks appeared between 2 and 13 min; in *B. unicolor*, peaks were observed from ~ 0.5 to 12 min, indicating a somewhat broader polarity range. In total, 26 distinct peptide sequences were identified: 9 in *L. carinatus* and 17 in *B. unicolor* (Tables 4 and 5). Three peptides—GRGAH, SGVGY and GGTHAW—were shared by both species. Most peptides were 5–7 amino acids long, with [M + H]^+^ masses between ~ 430 and 630 Da. Several sequences matched or resembled mucus peptides described in other gastropods^15^.

**Table 4:** Peptides identified in *L. carinatus* by LC–MS/MS analysis. Peptides were identified based on their MS/MS fragmentation patterns in comparison with reference data.

No	Rt (min)	Peptide sequence	[M + H]^+^ (m/z)	Main fragment ions (m/z)	References
1	2.09	GVSGN	432.23	375.09, 276.47, 190.39, 133.02	[13]
2	2.12	GPGSN	432.24	374.29, 276.15, 219.18, 133.04	
3	7.34	GRGAH	496.34	440.09, 283.19, 227.16, 155.28	
4	8.57	VATSF	524.38	425.23, 355.06, 253.10, 166.18	
5	8.84	GKTGY	526.44	468.32, 341.08, 239.03, 183.08	
6	8.89	SGVGY	482.37	395.37, 338.47, 239.03, 183.03	
7	9.4	GGTHAW	628.56	553.51, 496.06, 395.28, 257.26, 188.40	
8	9.5	APTAH	496.3	425.44, 328.63, 227.22, 157.14	
9	12.51	PGGNKR	628.42	531.42, 474.23, 417.39, 303.12, 177.03	

The table presents the retention time (Rt), peptide sequences, protonated molecular ions [M + H]^+^, and the corresponding major fragment ions (m/z) used for peptide identification.

**Table 5:** Peptides identified in *B. unicolor* by LC–MS/MS analysis. Peptides were identified based on their MS/MS fragmentation patterns in comparison with reference data.

No	Rt (min)	Peptide sequence	[M + H]^+^ (m/z)	Main fragment ions (m/z)	References
1	0.86	DVNGGR	618.22	502.35, 403.38, 288.97, 232.60, 174.26	^13^
2	3.38	INNGH	554.24	441.24, 327.25, 213.03, 156.03	
3	6.25	GRGAH	496.36	440.0, 284.21, 227.26, 156.85	
4	7.03	SAFGY	544.25	457.37, 385.25, 239.99, 182.28	
5	7.05	SGVGY	482.37	395.61, 338.29, 239.16, 181.23	
6	7.28	ADVGM	508.37	421.28, 306.43, 207.08, 151.19	
7	8.01	GGDDW	548.44	474.33, 418.46, 302.68, 186.05	
8	8.21	GGVNPR	600.44	541.48, 484.28, 385.34, 272.07, 175.00	
9	8.33	AGSANR	574.44	503.40, 447.84, 360.27, 290.01, 174.31	
10	9.34	GGTHAW	628.5	554.44, 496.40, 394.99, 258.97, 187.85	
11	9.44	VSKGY	552.36	453.44, 367.27, 239.19, 182.17	
12	9.47	SPADY	552.34	465.34, 368.11, 297.13, 182.28	
13	9.75	KAATY	552.31	425.36, 354.92, 283.19, 181.24	
14	10	ITGVY	552.29	439.20, 338.14, 281.42, 182.05	
15	10.34	AGSRY	552.36	482.45, 425.29, 338.44, 182.32	
16	11.34	GGSVPR	571.68	515.40, 458.48, 371.16, 272.54, 175.11	
17	11.52	AGGHTR	598.65	527.48, 470.46, 413.29, 275.24, 174.95	

The table presents the retention time (Rt), peptide sequences, protonated molecular ions [M + H]^+^, and the corresponding major fragment ions (m/z) used for peptide identification.

## Discussion

The present study demonstrates that crude protein extracts from the freshwater snails *L. carinatus* and *B. unicolor* possess multiple bioactivities, including modest antioxidant enzyme activity, moderate antimicrobial and antibiofilm effects and selective cytotoxicity towards certain human cancer cell lines, and contain diverse small peptides that may underlie these effects.

The SOD, CAT and GST activities measured in both species were relatively low compared with those reported for several marine snails. For example, GST from the marine gastropod *Turbo radiatus* exhibited a specific activity of 194.4 U/mg protein^29^, while Cu–Zn SOD from the marine snails *Cellana rota, Tectus dentatus* and *Rapana venosa* reached about 520.7, 658.3 and 600.7 U/mg protein^2,4,30^. Marine species often experience pronounced oxidative challenges due to fluctuating salinity, high ultraviolet irradiation and variable pollutant exposure, which may favour the evolution of stronger enzymatic defenses. Freshwater snails such as *L. carinatus* and *B. unicolor* inhabit more stable osmotic environments and may rely more on non-enzymatic antioxidants, small thiols or other stress-response proteins, as suggested for other freshwater species^16,31^.

Despite low antioxidant enzyme levels, both freshwater snail crude protein extracts exhibited clear antimicrobial activity. The inhibition of *E. coli* and *S. aureus*, and the strong activity of *L. carinatus* against *C. albicans*, are in line with previous observations that snail tissues and secretions can target bacterial and yeast pathogens^12,32,33^. The lack of activity against *A. niger* suggests that the bioactive components present are more effective against yeasts than filamentous fungi, which is similar to patterns reported for terrestrial snail extracts^33^. In comparison with terrestrial snails such as *Cryptozona bistrialis*, whose protein hydrolysates produced much larger inhibition zones against *S. aureus* and *P. aeruginosa*^32^, the freshwater snails examined here produced comparatively smaller inhibition zones. Even so, their low MIC values (7.81–31.24 µg/mL against the test bacteria and *C. albicans*) point to a clear antibacterial and antifungal potency, even though these values remained higher than those of the reference drugs. This activity may also be ecologically meaningful, contributing to defense against common freshwater microbes.

The antibiofilm assays indicate that both crude protein extracts can reduce biofilm formation, especially in *B. subtilis* and *P. aeruginosa*, albeit to a lesser extent than conventional antibiofilm drugs. Because biofilms confer tolerance to antibiotics and environmental stress, even partial inhibition may be advantageous in nature. Similar moderate antibiofilm effects have been reported for marine fungal metabolites and other invertebrate extracts^1,24^. The mechanisms may involve interference with initial adhesion, disruption of extracellular polymeric substances or modulation of quorum-sensing systems.

The cytotoxicity results are particularly noteworthy because they reveal pronounced selectivity. *L. carinatus* crude protein extract was strongly cytotoxic to MCF-7 and HeLa cells but essentially inactive against HCT-116, PC3 and HepG2, whereas *B. unicolor* crude protein extract was very potent against HCT-116 and moderately active against PC3 while sparing other cancer lines. Crucially, both crude protein extracts were considerably less toxic to normal fibroblasts than to their most sensitive cancer targets. This pattern resembles other studies in which snail-derived compounds showed preferential effects on tumor cells with limited toxicity for normal cells^9,34,35^. By comparison, doxorubicin displayed strong but non-selective toxicity, with similar IC_50_ values for cancer and normal cells.

The basis of this selectivity remains to be elucidated. Cancer cells often display altered membrane composition, dysregulated redox status and defective apoptosis pathways, which can be exploited by bioactive peptides and proteins. The peptide profiles identified here provide candidates that may contribute to the observed cytotoxicity. Many of the peptides are short, with sequences that could allow membrane interaction or intracellular penetration. In silico analyses of mucus peptides from terrestrial snails predicted anticancer properties for some short sequences^14,15^. It is plausible that certain peptides in *L. carinatus* and *B. unicolor* crude protein extracts interact selectively with tumour cell membranes or intracellular targets, inducing apoptosis or disrupting vital signaling pathways. The different cytotoxic spectra of the two species suggest that their peptide repertoires and other constituents differ enough to target distinct cancer types.

LC–MS/MS revealed both shared and species-specific peptides. The three common peptides (GRGAH, SGVGY and GGTHAW) may represent conserved motifs derived from ubiquitous proteins; their presence in both extracts suggests fundamental roles, perhaps in basic cell processes. In contrast, unique peptides such as GVSGN, GKTGY or GGSVPR in *L. carinatus* and DVNGGR, GGDDW or AGGHTR in *B. unicolor* may underpin the species-specific antimicrobial and cytotoxic activities. Although the present study did not functionally test individual peptides, the correlation between peptide diversity and bioactivity warrants further work. Synthesis and direct testing of these sequences could reveal new antimicrobial or anticancer peptides, as has been achieved for other molluscan peptides such as kahalalide F from a marine opisthobranch^36^.

This study has some limitations. First, the use of crude protein extracts means that activities cannot yet be attributed to specific molecules. Second, mechanistic assays (for example, apoptosis markers, membrane integrity or ROS measurements) were not performed, so the exact modes of action remain speculative. Third, all assays were in vitro; in vivo efficacy, stability and toxicity must be evaluated before any therapeutic application. Nevertheless, these findings establish that *L. carinatus* and *B. unicolor* are candidate freshwater sources of bioactive proteins and peptides with antibacterial, antibiofilm and selective anticancer properties.

## Conclusion

Crude protein extracts from the freshwater snails *Lanistes carinatus* and *Bellamya unicolor* exhibit modest antioxidant enzyme activities but clear antimicrobial, antibiofilm and selective anticancer effects in vitro. *L. carinatus* shows comparatively stronger antifungal and cytotoxic activity against MCF-7 and HeLa cells, whereas *B. unicolor* is highly active against HCT-116 colorectal cancer cells. Both extracts display only weak toxicity toward normal fibroblasts, suggesting a favorable therapeutic window relative to doxorubicin. LC–MS/MS analysis reveals a rich mixture of small peptides, including species-specific sequences that may underpin the observed biological activities. These results emphasize the potential of non-schistosome freshwater snails as reservoirs of bioactive compounds and provide a foundation for future studies aimed at isolating, characterizing and mechanistically evaluating individual snail-derived molecules as candidates for antimicrobial and anticancer applications.

## Supplementary Information

Below is the link to the electronic supplementary material.

Supplementary Material 1

## Data Availability

Data is provided within the manuscript files.
