# Supplementary figures and images for: Therapeutic potential of crude protein extracts from two Egyptian freshwater snails Lanistes carinatus and Bellamya unicolor
Source: Sci Rep. 2026 Jul 4;16:20554. doi: 10.1038/s41598-026-60044-5 (PMC13332020; doi:10.1038/s41598-026-60044-5)

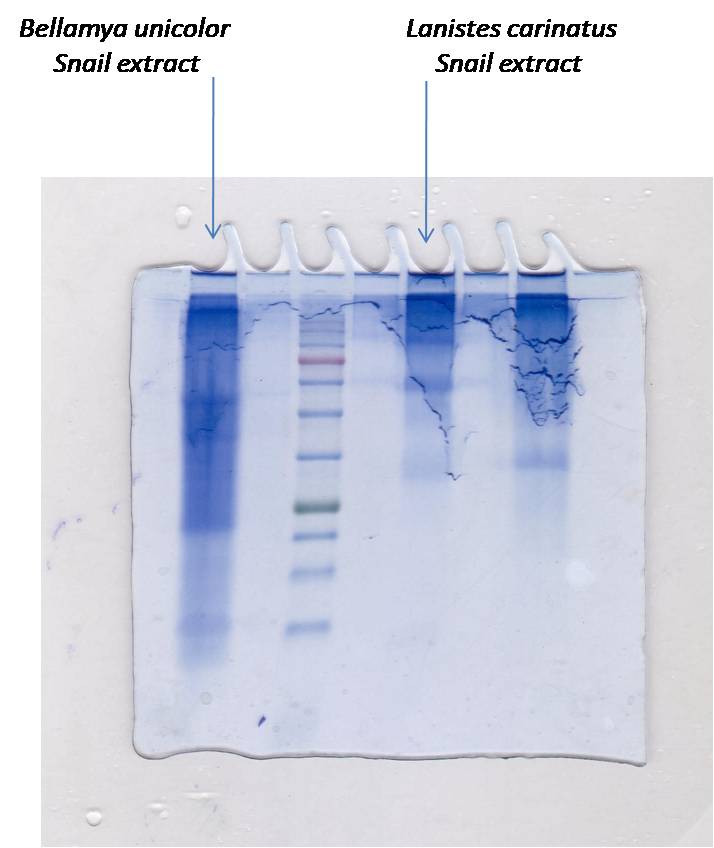

Supplement: Supplementary file 1 — Supplementary Material 1 [file 41598_2026_60044_MOESM1_ESM.jpg]
